# Setting Up an Ultra-Fast Next-Generation Sequencing Approach as Reflex Testing at Diagnosis of Non-Squamous Non-Small Cell Lung Cancer; Experience of a Single Center (LPCE, Nice, France)

**DOI:** 10.3390/cancers14092258

**Published:** 2022-04-30

**Authors:** Marius Ilié, Véronique Hofman, Christophe Bontoux, Simon Heeke, Virginie Lespinet-Fabre, Olivier Bordone, Sandra Lassalle, Salomé Lalvée, Virginie Tanga, Maryline Allegra, Myriam Salah, Doriane Bohly, Jonathan Benzaquen, Charles-Hugo Marquette, Elodie Long-Mira, Paul Hofman

**Affiliations:** 1 Laboratory of Clinical and Experimental Pathology, Pasteur Hospital, Université Côte d’Azur, 06000 Nice, France; ilie.m@chu-nice.fr (M.I.); hofman.v@chu-nice.fr (V.H.); bontoux.c@chu-nice.fr (C.B.); lespinet-fabre.v@chu-nice.fr (V.L.-F.); bordone.o@chu-nice.fr (O.B.); lassalle.s@chu-nice.fr (S.L.); lalvee.s@chu-nice.fr (S.L.); long-mira.e@chu-nice.fr (E.L.-M.); 2 Biobank-related Hospital (BB-0033-00025), Pasteur Hospital, 06000 Nice, France; tanga.v@chu-nice.fr (V.T.); allegra.m@chu-nice.fr (M.A.); salah.m2@chu-nice.fr (M.S.); bohly.d2@chu-nice.fr (D.B.); 3 FHU OncoAge, Pasteur Hospital, Université Côte d’Azur, 06000 Nice, France; benzaquen.j@chu-nice.fr (J.B.); marquette.c@chu-nice.fr (C.-H.M.); 4 Inserm U1081, CNRS UMR 7413, IRCAN, 06100 Nice, France; 5 Department of Thoracic/Head and Neck Medical Oncology, MD Anderson Cancer Center, Houston, TX 77030, USA; SHeeke@mdanderson.org; 6 Department of Pulmonary Medicine and Thoracic Oncology, Pasteur Hospital, 06000 Nice, France

**Keywords:** genomic alteration, next-generation sequencing, turnaround time, non-squamous non-small cell lung carcinoma, targeted therapy

## Abstract

**Simple Summary:**

Due to the increase of molecular biomarkers to be characterized to tailor therapeutic strategies for non-squamous non-small cell lung carcinoma (NS-NSCLC), it is now more and more challenging to evaluate these biomarkers using sequential analyses. We report here our experience concerning the development of an optimal workflow for genomic alteration assessment as reflex testing in routine clinical practice at diagnosis for NS-NSCLC patients by using an ultra-fast-next generation sequencing (NGS) approach. The analytical validation of the NGS workflow demonstrated 100% concordance with the gold standard methods. Only few cases failed for DNA/RNA NGS results. The mean turnaround time (TAT) was 72 h (ranging from 48 to 96 h). An ultra-fast NGS technique can maximize the management of the sample workflow in thoracic oncology to obtain the molecular biology results in an appropriate TAT, even when only a small amount of nucleic acids is available.

**Abstract:**

The number of genomic alterations required for targeted therapy of non-squamous non-small cell lung cancer (NS-NSCLC) patients has increased and become more complex these last few years. These molecular abnormalities lead to treatment that provides improvement in overall survival for certain patients. However, these treated tumors inexorably develop mechanisms of resistance, some of which can be targeted with new therapies. The characterization of the genomic alterations needs to be performed in a short turnaround time (TAT), as indicated by the international guidelines. The origin of the tissue biopsies used for the analyses is diverse, but their size is progressively decreasing due to the development of less invasive methods. In this respect, the pathologists are facing a number of different challenges requiring them to set up efficient molecular technologies while maintaining a strategy that allows rapid diagnosis. We report here our experience concerning the development of an optimal workflow for genomic alteration assessment as reflex testing in routine clinical practice at diagnosis for NS-NSCLC patients by using an ultra-fast-next generation sequencing approach (Ion Torrent Genexus Sequencer, Thermo Fisher Scientific). We show that the molecular targets currently available to personalized medicine in thoracic oncology can be identified using this system in an appropriate TAT, notably when only a small amount of nucleic acids is available. We discuss the new challenges and the perspectives of using such an ultra-fast NGS in daily practice.

## 1. Introduction

Thanks to the availability of different targeted therapies for treatment of late-stage non-squamous non-small cell lung carcinoma (NS-NSCLC), depending on some of the genomic alterations, the overall survival (OS) of these patients has improved in recent years [1,2]. In this respect, the number of mandatory genes for analysis and of molecular targets to look for when treating NS-NSCLC has increased progressively over recent years and requires tissue biopsies, cytological specimens, and in certain situations, liquid biopsies [3,4,5,6]. Therefore, it is mandatory today, according to the recent international guidelines, to systematically obtain, at diagnosis, the status of *EGFR*, *ALK*, *ROS1*, *BRAF*, *NTRK*, *RET*, and *MET*, for late-stage NS-NSCLC treatment [5,7].

Moreover, since new drugs have recently given promising results in clinical trials, many genomic alterations will soon be searched for in a number of other genes, notably *KRAS* and *HER2*, but also *NRG1* and *NUT* [3,8]. Knowing the status of different associated genes, such as *STK11*, *KEAP1*, and *TP53*, may also be of strong interest in the future for therapeutic strategies for these patients, to better predict the response or resistance to some targeted therapies and/or immune checkpoint inhibitors (ICIs) [9,10,11,12].

ICIs alone or in association with chemotherapy is now the standard of care for late-stage NS-NSCLC provided that these tumors do not have molecular alterations accessible to a targeted therapy [13,14]. Moreover, the upcoming development of neoadjuvant and adjuvant immunotherapy will lead to systematically look for genomic alterations in *EGFR* and *ALK* (at least) in early-stage NS-NSCLC, since ICIs should be administered only in *EGFR* and *ALK* wild-type tumors [15]. Moreover, it is now mandatory to look for *EGFR* mutations in early-stage NS-NSCLC, since a third-generation EGFR tyrosine kinase inhibitor (osimertinib) can be given as adjuvant therapy in some *EGFR* mutated tumors [16,17].

In this regard, the strategy for assessing the different biomarkers that can predict the response to the different targeted therapies and/or immunotherapies for NS-NSCLC patients is moving fast, making it increasingly difficult to identify these biomarkers in a sequential manner [7,18,19]. The association of next-generation sequencing (NGS) (for genomic alteration evaluation) and immunohistochemistry (IHC) (at least for PD-L1 status assessment) approaches is now the best way to obtain information concerning all the mandatory therapeutic targets [4,5,20]. However, these analyses need to take into consideration the sample size and the amount of extracted nucleic acids, and should be done in accordance with the TAT to obtain the results [4,5,21,22].

Here, we describe the experience of a single center (Laboratory of Clinical and Experimental Pathology Laboratory (LPCE), Nice University Hospital, France) in setting up an ultra-fast DNA and RNA-based NGS assay for molecular biology reflex testing at diagnosis of NS-NSCLC.

The advantages and the limitations of this workflow from the tissue biopsy to the report are discussed. Finally, we provide details into the current challenges of biomarker assessment of NS-NSCLC and a few perspectives related to this domain.

## 2. Materials and Methods

### 2.1. Patients

This cohort included 345 consecutive patients diagnosed with NS-NSCLC between 20 September and 31 January 2022. The patients had a fast DNA- and RNA-based NGS as reflex testing at the LPCE (Nice University Hospital, France), which is accredited according to the ISO 15189 norm for somatic genomic testing by NGS in routine clinical practice (www.cofrac.fr, accessed on 22 February 2022).

All tumor specimens were used with the informed signed consent from the patients. The study was approved by the local ethics committee (Human Research Ethics Committee, Nice University Hospital Center/Hospital-related Biobank BB-0033-00025; http://www.biobank-cotedazur.fr/, accessed on 18 January 2022) and was performed in accordance with the guidelines of the Declaration of Helsinki.

### 2.2. Ultra-Fast Next-Generation Sequencing

**Figure 1:**
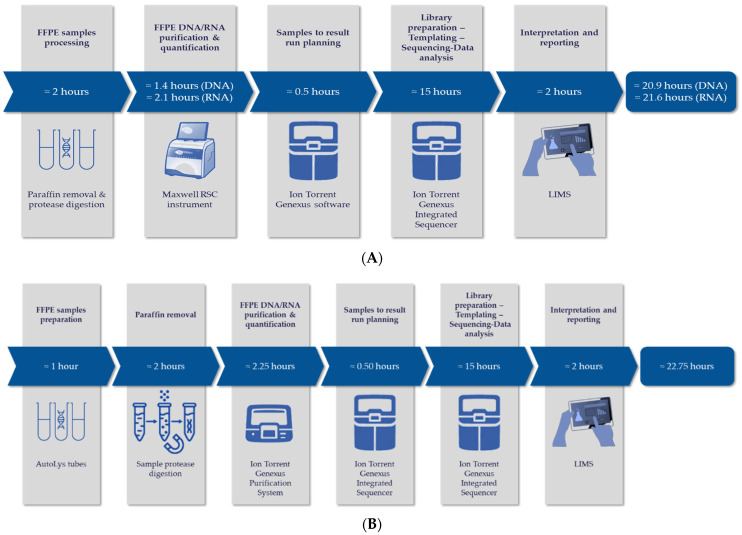
Genexus System workflows used in this study. (**A**) Workflow using the Maxwell automate for DNA and RNA extraction. (**B**) Workflow with AutoLys tubes and Genexus Purification System for DNA and RNA extraction.

Briefly, nucleic acids were either extracted using the Maxwell RSC Instrument (Promega, catalog number AS4500) with the Maxwell RSC FFPE Plus DNA kit (catalog number AS1720) and Maxwell RSC RNA FFPE kit (catalog number AS1440), or using the Ion Torrent™Genexus™Purification System (Thermo Fisher Scientific, Waltham, MA, USA; catalog number A48148) with the Genexus™ FFPE DNA/RNA Purification Combo Kit (Thermo Fisher Scientific, catalog number A45539) (Figure 1).

Following nucleic acids’ extraction with the Maxwell RSC Instrument automaton, a Qubit Fluorometric quantification assay (Thermo Fisher Scientific, catalog number Q33327) was performed with the Qubit RNA HS Assay Kit (catalog number Q32852) and Qubit dsDNA HS Assay Kit (catalog number Q32851) to measure the concentration of extracted nucleic acid. The Ion Torrent™Genexus™ Purification System (Thermo Fisher Scientific) was equipped with a fluorometer and automatically assayed the extracted nucleic acid following the extraction step. Detection of genomic alterations was then performed using Ion semiconductor sequencing (Ion Torrent™ Technology, Thermo Fisher Scientific) on the Ion Torrent™Genexus™Integrated Sequencer. The panel used was the Oncomine™ Precision Assay GX (OPA, Thermo Fisher Scientific, catalog number A46291). This panel included 50 key genes of which 45 were targeted for DNA mutation detection, 18 for fusion detection and 14 for Copy Number Variant (CNV) detection. The panel also incorporated a 5′/3′ expression imbalance caller for the detection of novel fusions. With this panel, the Genexus sequencer was able to sequence up to 16 samples on a single run.

The TAT was considered as the time between obtaining the formalin fixed paraffin embedded (FFPE) sample and the electronic validation of the NGS report with the hospital software. This time included the deparaffinization step, the nucleic acids’ extraction steps, the sequencing step, the genomic alteration(s) assessment, and the post analytical time (Figure 1).

The performance of the Genexus workflow was assessed by comparison to the following gold standard methods used at the LPCE before starting this new workflow: (i) Idylla *EGFR* Mutation Test (Biocartis, Mechelen, Belgium); (ii) Idylla *KRAS* Mutation Test (Biocartis); (iii) ALK IHC (clone D5F3, Roche-Ventana, Tucson, AZ, USA) and/or *ALK* fluorescence in situ hybridization (FISH) (Vysis *ALK* Break Apart FISH Probe Kit, Abbott Molecular, Des Plaines, IL, USA); (iv) ROS1 IHC (clone D4D6, Cell Signaling Technology, Danvers, MA, USA) and/or FISH (POSEIDON *ROS1* Dual Color Break Apart Probe, Leica Biosystems, Amsterdam, The Netherlands); (v) BRAFV600E IHC (clone VE1, Roche-Ventana), and (vi) the S5 system (Thermo Fisher Scientific) using the DNA Ion AmpliSeq™Cancer Hotspot Panel and the RNA Oncomine Focus Assay.

## 3. Results

In this study, 345 NSCLC specimens were examined at the LPCE between the period study. Among these tumors, 259/345 (75%) corresponded to NS-NSCLC and underwent clinical ultra-fast NGS testing. The main epidemiological, clinical, and pathological data are shown in Table 1.

Two expert thoracic pathologists (V.H. and M.I.) blindly assessed the percentage of tumor cells and any discrepancy was discussed using a multihead microscope. Samples with more than 10% of tumor cells were selected for nucleic acid extraction and further NGS analyses depending on the amount of nucleic acid extracted with a cutoff of 13 ng of DNA or RNA.

The majority of tests were performed on in-house samples, 248 (96%), with 11 (4%) performed on samples from outside centers.

Overall, 506 NGS analyses were performed (Table 2). Ten cases (4%) failed for DNA NGS results due to a low quality of nucleic acids (6 cases) and a low amount of nucleic acid (4 cases <13 ng of DNA, whereas 5 cases (2%) failed for RNA NGS results due to the poor quality of RNA and low amount of RNA (<13 ng of RNA). The mean % of tumor cells in the failed cases was low (15%, range 10% to 20%), whereas the range % of tumor cells in successful cases was between 30% to 90%. Of the 15 failed specimens, 11 were small tissue biopsies (mean area, 2 mm^2^), while 4 were cytological specimens (e.g., endobronchial ultrasound (EBUS)).

**Figure 2:**
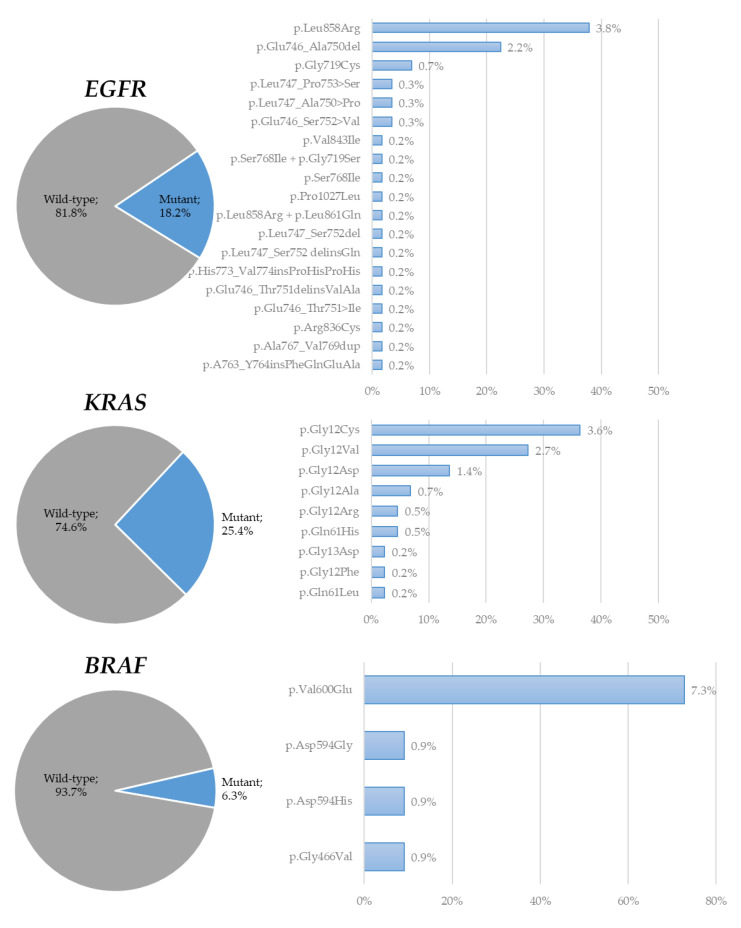

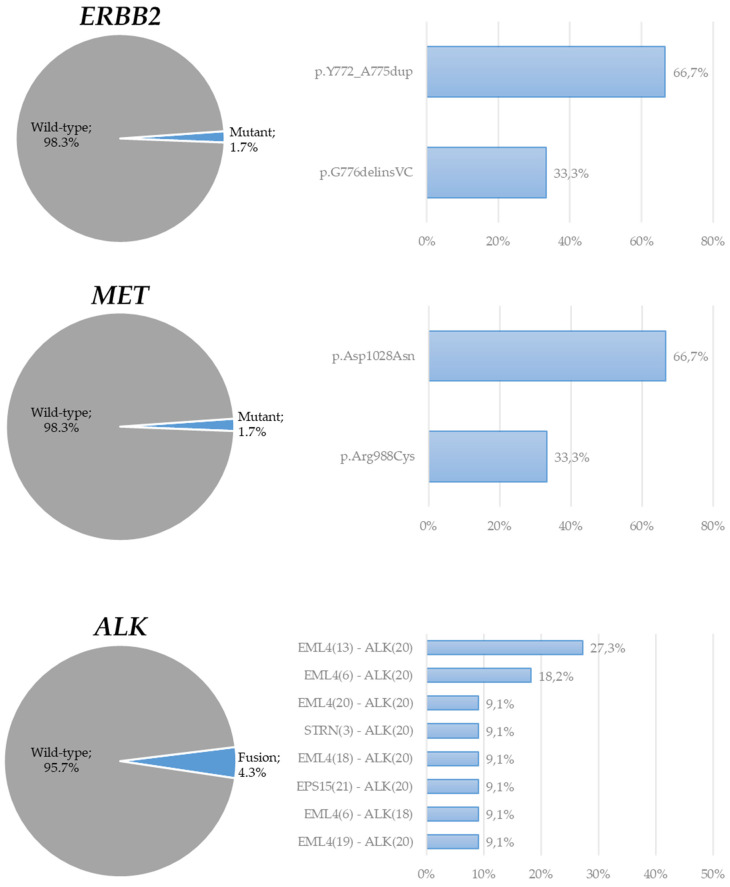

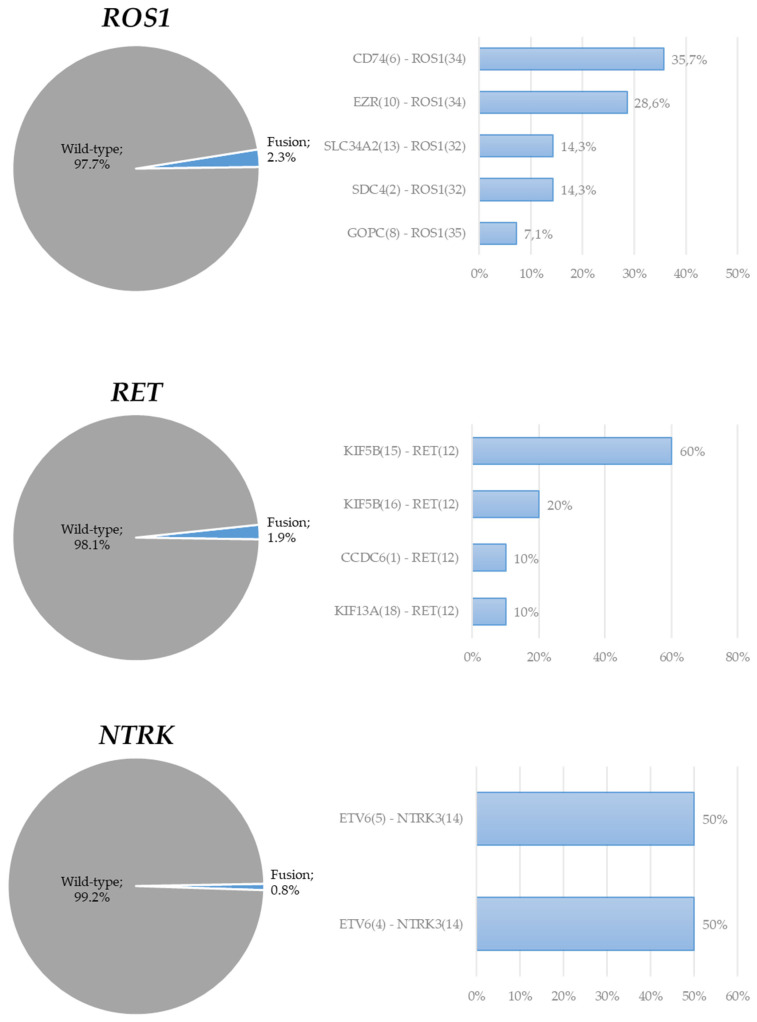
Distribution of driver molecular alterations detected in NS-NSCLC specimens during the study period.

Of the 259 patients with NS-NSCLC with valid or available NGS analysis (*n* = 242), driver molecular alterations were detected as follows (Figure 2): EGFR, 44 (18.2%); KRAS, 61 (25.4%), BRAF, 15 (6.3%); ALK 10 (4.3%); ERBB2, 4 (1.7%); MET, 4 (1.7%); ROS1 11 (2.3%); RET, 9 (1.9%), and NTRK, 2 (0.8%), with additional alterations detected with an incidence below 1% (e.g., IDH1, CDKN2A, FGFR3, KIT, MTOR, FGFR4. NTRK3, NRAS, PIK3CA, IDH2, ERBB3, ERBB4, AR, CHECK2, SMO, PTEN).

The mean TAT was 72 h (range, 48 to 96 h) for all specimens, including those referred in from outside clinical centers.

The analytical validation of the Genexus OPA DNA RNA workflow demonstrated 100% concordance with the gold standard methods, as follows: (i) the DNA S5 Hotspot panel, 14 cases (EGFR, 4; KRAS, 2; BRAF, 1; PIK3CA, 1; TP53, 10; KIT, 3); (ii) the RNA Oncomine Focus Assay panel, 22 cases (ALK, 4; ROS1, 1; FGFR3, 1; RET, 1; MET, 3); (iii) the Idylla EGFR Mutation Test (*n* = 109, wild-type, 61, and mutated, 48); (iv) the Idylla KRAS Mutation Test (*n* = 30, wild-type, 23, and mutated, 7); (v) BRAFV600E IHC (*n* = 107, wild-type, 99; and mutated, 8); (vi) IHC and/or FISH ALK (*n* = 65; wild-type, 60, and rearranged, 5), and (vii) IHC and/or FISH ROS1 (*n* = 65; wild-type, 60; rearranged, 5).

## 4. Discussion

### 4.1. Advantages and Limitations of a Fast-Next Generation Sequencing

#### 4.1.1. Advantages

The usefulness of ordered testing for molecular biomarkers for lung adenocarcinomas has been highlighted by many studies [23,24]. An NGS approach can optimize the management of the sample workflow in thoracic oncology to obtain the molecular biology results. Thus, NGS testing of advanced NS-NSCLC directly affects patient OS [23,25]. In this context, it has been demonstrated that NGS reflex testing for advanced NS-NSCLC is cost-effective. Reliable *in-house* molecular testing systems, including the alterations for all Food and Drug Administration (FDA) and European Medicine Agency (EMA) approved and international guideline-recommended therapies, as well as other emerging markers for these tumors are available [5,26,27]. The genomic alterations that, to date, can be detected on the different genes present in the OPA, DNA, and RNA panels cover the great majority of targets that can be associated with the current drugs for NS-NSCLC treatment [5]. An ultra-fast NGS is of strong interest since many of the NGS approaches described up to now can have a TAT to obtain the results that are sometimes not compatible with urgent therapeutic care leading to the use in parallel of rapid genotyping based on a RT-PCR approach [28,29]. To save time, notably for certain NSCLC patients such as non-smokers, some strategies use, in front-line, a frozen specimen to avoid the time due to the usual pre-analytical phase preformed in a pathology laboratory (formalin fixation and paraffin embedding steps). When using an ultra-fast NGS, the thoracic oncologist can obtain in around three working days, the “molecular portrait” of the tumor. Moreover, these molecular results can be obtained almost simultaneously from the IHC results, notably those concerning the percentage of PD-L1 positive tumor cells [20]. For this, the physician has the assessment of all the biomarkers in hands for the best therapeutic choice of first-line treatment, to give a targeted therapy or an immuno/immuno-chemotherapy [30]. More exceptionally, the patient can be included into a clinical trial depending on the genomic and/or the PD-L1 IHC results [31,32,33,34]. Depending on the use of the DNA OPA panel alone or in combination with the RNA OPA panel, and on the systematic use of a control sample, the number of tumors that can be analyzed on each run can vary from 7 to 16. Knowing that, at least in theory, it is possible to make three runs per week, and thus, 48 patients with tumors can benefit from this ultra-fast NGS analysis weekly.

The Genexus sequencer and, even more, the combination of this device and the nucleic acid Genexus purification system, reduces the time to obtain the results and the amount of work of the technicians. The possibility of quantifying the extracted DNA and RNA before library preparation, as well as the capacity to use low amounts of extracted nucleic acid (from a minimum of 13 ng of DNA or 13 ng of RNA, according to the specifications) allows rapid evaluation of the feasibility of the sequencing analyses. The possibility of using a low amount of nucleic acid is pivotal, due to the number of biopsies of very small size in thoracic oncology and also since some of the profiles of genomic alteration sometimes need to be performed with cytological samples [35,36,37].

Due to the progressive increase in the number of molecular biomarkers to be characterized to tailor therapeutic strategies for NS-NSCLC, it is now more and more challenging to evaluate these biomarkers using sequential analyses, which can involve, depending on the gene of interest, immunohistochemistry, FISH, and/or RT-PCR [38,39,40,41]. Moreover, these sequential analyses have the disadvantage of rapid exhaustion of the sample and, depending on the number of genes to be evaluated can be costly [42]. Conversely, analyses using NGS are cost-effective and overall allow the biological material to be saved [38,42,43,44,45,46,47,48,49,50]. However, for this approach, it is necessary to run enough samples at the same time to save on consumables, which is sometimes not possible, depending on the patient recruitment and the molecular pathology laboratory [51].

#### 4.1.2. Limitations

The development of an ultra-fast NGS tool as reflex testing at diagnosis of NSCLC, as described above, may hold certain limitations in daily practice. First, the number of genes available on the OPA panel (50 genes) is much lower than those present on large panels containing several hundreds of genes [52]. However, the OPA panel includes all the genes that are currently mandatory to detect a genomic alteration that can be associated with a current targeted therapy in thoracic oncology. It is noteworthy that some genes of interest in thoracic oncology (such as *KEAP1*, *Rb1*, and *SMARCA4*) were not present on the OPA panels used in the present work. Thus, some additional genes might be useful to evaluate prognosis or to predict response to some targeted therapies and immunotherapies [12,53].

The possibility of missing some partners of gene fusions or some exceptional mutations is theoretically possible when using ultra-fast NGS due to the amplicon sequencing technology [54].

The systematic use of an NGS approach for genomic alteration assessment of NSCLC at diagnosis as reflex testing could be considered as not very effective in comparison to performing an NGS approach only in case of physician bespoke testing. Thus, some testing can be done on an early-stage NSCLC for which, aside from osimertinib adjuvant treatment of *EGFR* mutated NS-NSCLC patients, no targeted therapies are currently available in routine daily practice. However, this practice raises questions related to the economic model and to the reimbursement of the NGS in general, which can be problematic depending on the country, notably in Europe [55]. Moreover, the reimbursement of NGS for early-stage NSCLC as well as the possibility of generating an unnecessary additional workload for the staff of the laboratory should be discussed. However, to systematically have, at the same time, the molecular profile of the early-stage NS-NSCLC, can be of strong interest when tumor recurrence or progression occurs. Thus, this can avoid doing an invasive re-biopsy at tumor progression to do some molecular biology testing. Moreover, the decision to provide targeted therapy can potentially be rapid at tumor progression without doing molecular biology testing on an FFPE block, which usually would be available in the pathology laboratory archives but for which the tissue may be exhausted. As mentioned above, the cost-effectiveness of NGS also depends on the number of samples available for each run, and should not just be used for *EGFR*, *ALK*, and *ROS1* assessment, but to look for all the mandatory biomarkers of NS-NSCLC stipulated in the international guidelines [56,57]. The simultaneous use of both DNA and RNA NGS, although ideal, is debatable. An alternative strategy would be to do a first-step DNA NGS, and in the case of no detection of genetic alterations to do an RNA NGS in a second step to look, for example, for a gene fusion, knowing that most of the genomic alterations for a targeted therapy are mutually exclusive [38]. However, this reflex strategy would consequently lead to a longer TAT to obtaining a report.

The quantity as well as the quality of extracted nucleic acids can be one of the limits for the robustness of NGS analyses. This is particularly the case when using hybrid capture sequencing technology and large panels [8]. In this respect, the Genexus sequencing technology and the OPA panels offer the advantage of requiring only, at least, 13 ng of DNA and RNA. This is highly valuable in thoracic oncology since, currently, the size of samples are becoming smaller and smaller [58,59,60]. In our study, the overall failure rate was low (15/506, 2%), despite a high number of small samples (biopsies, 77% and cytology 8%). However, the failed specimens were notably small-sized specimens with low percentage of tumor cells (<10%). When dealing with a failed analysis, the oncologist should be consulted to examine the possibility of a tissue re-biopsy, according to the patient status and/or to the tumor accessibility, or of a liquid biopsy for making NGS from circulating free DNA [61].

### 4.2. Current Challenges Associated with the Rapid Revolution of Predictive Biomarkers in NS-NSCLC

The current mandatory biomarkers to look for in late-stage NS-NSCLC include different genomic alterations present on *EGFR*, *ALK*, *ROS1*, *NTRK*, *BRAF*, *MET*, and *RET*, as well as the PD-L1 expression of tumor cells [4,5]. The presence or absence of one of these biomarkers leads to the possibility of proposing targeted therapy or immunotherapy alone or combined with chemotherapy [4,7,18,30,51]. Therefore, different therapeutic molecules have obtained authorization from the FDA in the USA and the EMA in Europe for first-line treatment in daily practice [7,18]. Other molecular alterations can be examined on *HER2*, *KRAS*, and the *EGFR* exon 20 mutation that can lead to patient inclusion, when not showing targetable abnormalities on the previous cited genes, in clinical trials [62,63,64]. More recently, combinations of different therapies including targeted treatment and immunotherapy are ongoing in clinical trials, and might be proposed in the near future as first-line treatment or on tumor progression [65,66]. It is noteworthy that all these therapeutic strategies are mainly indicated to date for NS-NSCLC histological subtypes. So, most of the different treatments for squamous cell (SC)-NSCLC are based on immunotherapy associated or not with chemotherapy [67,68]. More exceptionally, a genomic alteration in one of the previous cited lists of genes can be looked for in young and/or nonsmoking SC-NSCLC patients or on the physician’s request, and can be associated with a targeted therapy [69,70,71]. The biomarkers associated with these different personalized treatments are mainly looked for on tissue biopsies, or sometimes on cytological samples [35]. Exceptionally, a liquid biopsy can be performed at baseline to look for some genomic alterations in case a tissue biopsy cannot be done or if the tissue sample cannot provide good quantity and/or quality nucleic acid for biomarker testing [61,72,73]. Despite the progress in surgical technologies and approaches to neoadjuvant or adjuvant chemotherapies or radio-chemotherapies, early-stage NSCLC patients still have a poor prognosis as they are associated with a death rate of around 50% of patients following complete tumor resection [74,75]. In this regard, the recent emergence of different targeted therapies and of immunotherapies for these patients is opening promise for better care and improvement in OS [76,77,78,79]. These new therapeutic approaches may be administered in neoadjuvant or adjuvant settings and need to be adapted according to the biomarkers identified by molecular testing and immunohistochemistry [80]. Adjuvant therapy with osimertinib for *EGFR* mutated NS-NSCLC (exon 19 deletion or p.L858R mutation) has received FDA and EMA approval for resected stage IB-IIIA, and thus can be used in daily practice [17]. Other therapeutic strategies, such as neoadjuvant immunotherapy in wild-type *EGFR* and *ALK* NS-NSCLC can, to date, be only discussed in clinical trials [81,82]. Moreover, many other clinical trials are currently ongoing for different targeted therapies for *ALK* rearrangements or adjuvant immunotherapies in early-stage NS-NSCLC [81,83]. All these treatments require identification of an associated biomarker that is characterized in tissue biopsies, cytological samples or in surgical resected specimens [35,72]. However, two challenges must be mastered: first, the TAT to obtain the results that are compatible with the therapeutic strategies, and secondly, the quality of the specimen to perform the different analyses [35,72]. Therefore, it is mandatory to adapt the best practices for effective partnerships and improve collaboration among thoracic surgeons, oncologists, pathologists, and other members of the multidisciplinary lung cancer care team who are managing patients with early-stage NSCLC. In this context, an ultra-fast NGS approach is certainly the best way to control all the different biomarkers that could be associated now and in the future with optimal treatment for early-stage NSCLC.

Finally, considering that the tumor stage at diagnosis is rarely known by the pathologist when examining the tumor tissue section with a microscope, one of the best strategies to optimize NS-NSCLC patient care, is to ask systematically, in addition to at least PD-L1 IHC, for reflex NGS [24,26].

NGS of liquid biopsies in advanced NS-NSCLC provides a great opportunity to detect targetable genomic alterations, which may not always be present in a tissue biopsy, due to tumor heterogeneity [30]. Moreover, according to the health care organization, liquid biopsies, in addition to being non-invasive and repeatable, can provide rapid molecular results [84]. So, some studies highlight the concept of “plasma first” to obtain results for advanced NS-NSCLC [84]. However, this is debatable since characterization of the histological subtype, and PD-L1 evaluation, as well as the detection of some genomic alterations, such as amplification and gene fusion, is not possible or is less sensitive with blood than with tissue samples [8,61]. However, to combine at diagnosis NGS with a liquid and a tissue biopsy could be, at least in theory, of strong value to detect all the current biomarkers of interest in late-stage NS-NSCLC, notably due to tumor heterogeneity [85].

### 4.3. What Should We Expect in the Future?

When thinking of the implementation of NGS as reflex testing at diagnosis of NS-NSCLC patients, we need to consider the biomarkers currently mandatory in practice, in addition to those that may be asked for when using targeted therapies in development (such as *NRG1* and *NUT*), and for future new therapies [8]. It is certain that a medium- sized NGS panel containing 50 genes, including the main genes of interest in thoracic oncology, currently allows identification of the different genomic alterations that can be associated with one of the targeted therapies available today. However, it is probable that, since many new targets will certainly be associated with different drugs in the future, a large panel (containing several hundreds of genes) will be essential in the near future [51]. Currently, large panels can be used in some circumstances when patients are included in clinical trials since rare genomic alterations detected in some specific genes are not present in medium-sized gene panels [86]. So, the role of a tumor board in evaluating the best treatment according to complexity of genomic alteration assessment will become more and more indispensable [87,88].

The assessment of different protein biomarkers (not only for patient diagnosis, but also for the prediction of response to treatment) takes advantage of multiplex IHC approaches. By evaluating a variable number of proteins at the same time on one tissue section, this technology saves tissue specimens. In a routine clinical practice, it is probable that chromogenic multiplex IHC is an easy approach for surgical pathologists to set up in their daily work [89,90]. So, by combining multiplex IHC and NGS testing, the care of NSCLC patients will certainly be optimal at diagnosis.

Daily practice of liquid biopsies is, today, a useful approach at tumor progression to detect a resistance mechanism that can be treated with targeted therapy [73]. At diagnosis of advanced NS-NSCLC, as mentioned above, the use of NGS, both with liquid and tissue biopsies may be of strong interest to optimize the maximal detection of a drug targetable to genomic alteration [28,84]. For early-stage NS-NSCLC, liquid biopsies will certainly be implemented in the future in routine practice to detect early recurrence after surgery [91]. Therefore, quantifying the circulating free tumor DNA before and at different time points after surgery will become a biomarker of patient prognosis [92]. Moreover, the future optimization of nucleic acid extraction and characterization from plasma samples may allow selection of a liquid biopsy at baseline to identify different molecular abnormalities for better care, even if the amount of tumor DNA shedded into blood is usually low in early-stage NS-NSCLC [93,94].

To date, many considerations for personalized medicine have been made for NS-NSCLC treatment. Therefore, minimal targeted therapy for squamous cell (SC)-NSCLC is currently available since no molecule has yet been FDA and EMA approved for this histological subtype. However, promising research and some innovative molecules are in the pharmaceutical pipelines that open up room for new SC-NSCLC treatments in the near future [95]. In this regard, it will certainly be necessary to ask for NGS reflex testing at diagnosis of advanced SC-NSCLC when new drugs are used in clinical trials and then in daily practice.

Targeted NGS may provide in the future valuable information to predict recurrence and identify patients at a high risk of recurrence. This could facilitate selection of the treatment strategy with close monitoring and adjuvant-targeted therapy [96].

## 5. Conclusions

Ultra-fast NGS integration as reflex testing may be an optimal option for genomic alteration assessment at diagnosis of all stage NS-NSLC. This strategy allows sensitive and specific evaluation of the status of 50 genes, looking for mutations, fusions, and amplifications when using a low amount of nucleic acid. When a targeted therapy or an immunotherapy has to be rapidly administered, the absence of initial selection of an analysis by sequencing, depending on the tumor stage, and without waiting for the physician’s request saves time for the patient. It is quite certain that many targeted therapies will probably soon be made available for early-stage NS-NSCLC treatment in routine clinical practice, reinforcing the necessity to look for different molecular alterations at the time of diagnosis. This leads unavoidably to the use of an ultra-fast NGS approach at diagnosis in this setting. Moreover, it will be of added value to combine ultra-fast NGS and multiplex IHC approaches for the identification of the histological classification with both genomic and proteomic biomarkers obtained at the same time [20,89,90]. The latter in situ technologies will allow tissue samples to be saved for further NGS development.

## Figures and Tables

**Table 1:** Main epidemiological and clinico-pathological features of the study population. EBUS: endobronchial ultrasound.

Characteristics		*n* (%)
		259 (100)
**Age at diagnosis**	Median	69
	Range	34–84
**Gender**	Male	197 (76)
	Female	62 (24)
**Smoking status**	Smokers	186(72)
	Non-smokers	44 (17)
	Unknown	29 (11)
**Type of specimens**	Bronchial biopsy	153 (59)
	Transthoracic biopsy	47 (18)
	Surgical specimen	39 (15)
	Pleural effusion (cellblock)	13 (5)
	EBUS	7 (3)
**Histology**	Adenocarcinoma	250 (97)
	Large cell carcinoma	9 (3)
**pTNM stage**	Stage I	78 (30)
	Stage II	73 (28)
	Stage III	28 (11)
	Stage IV	80 (31)

**Table 2:** Throughput of analyses during the study period. OPA: Oncomine Precision Assay.

**OPA analyses ** **Overall = 506**	DNA	252
	RNA	254
	Failed DNA	10/252 (4%)
	Failed RNA	5/254 (2%)

## Data Availability

Data are available on request.
